# Long-term quality of life in necrotizing soft-tissue infection survivors: a monocentric prospective cohort study

**DOI:** 10.1186/s13613-021-00891-9

**Published:** 2021-07-02

**Authors:** Tomas Urbina, Florence Canoui-Poitrine, Camille Hua, Richard Layese, Aline Alves, Rachida Ouedraogo, Romain Bosc, Emilie Sbidian, Olivier Chosidow, Armand Mekontso Dessap, Nicolas de Prost, Henri Mondor Hospital Necrotizing Fasciitis Group, Romain Bosc, Olivier Chosidow, Nicolas de Prost, Camille Hua, Raphaël Lepeule, Alain Luciani, Lionel Nakad, Françoise Tomberli, Tomas Urbina, Paul-Louis Woerther

**Affiliations:** 1 Médecine Intensive Réanimation, Hôpital Saint-Antoine, Assistance Publique-Hôpitaux de Paris (AP-HP), 75571 Paris Cedex 12, France; 2 Sorbonne Université, Université Pierre-Et-Marie Curie, Paris 6, France; 3 Service de Médecine Intensive Réanimation, Hôpitaux Universitaires Henri Mondor, Assistance Publique-Hôpitaux de Paris (AP-HP), Créteil, France; 4 Université Paris-Est Créteil Val de Marne (UPEC), Créteil, France; 5 Service de Santé Publique, Hôpitaux Universitaires Henri Mondor, Assistance Publique – Hôpitaux de Paris (AP-HP), Créteil, France; 6 Service de Dermatologie, Hôpitaux Universitaires Henri Mondor, Assistance Publique – Hôpitaux de Paris (AP-HP), Créteil, France; 7 Plastic, Reconstructive, Aesthetic and Maxillofacial Surgery Department, Henri Mondor Hospital, 51 Avenue du Marechal de Lattre de Tassigny, 94000 Créteil, France; 8 Henri Mondor Breast Center, Henri Mondor Hospital, 51 Avenue du Marechal de Lattre de Tassigny, 94000 Créteil, France; 9 Biology of the NeuroMuscular System, INSERM Team U955-E10, Paris East University, 94000 Créteil, France; 10 Centre D’Investigation Clinique 1430, Inserm, 94010 Créteil, France; 11 Groupe de Recherche Clinique CARMAS, Université Paris Est-Créteil, Créteil, France

**Keywords:** Necrotizing soft-tissue infection, Necrotizing fasciitis, Quality of life, Critical care, Septic shock, SF-36, Intensive care unit, Outcome

## Abstract

**Background:**

Compared to other life-threatening infection survivors, long-term health-related quality of life (QOL) of patients surviving necrotizing soft-tissue infections (NSTI) and its determinants are little known.

In this monocentric prospective cohort including NSTI survivors admitted between 2014 and 2017, QOL was assessed during a phone interview using the 36-Item Short-Form Health Survey (SF-36), the Hospital Anxiety and Depression (HAD), the activity of daily living (ADL), instrumental ADL (IADL) scales and the Impact of Event Scale-Revised (IES-R). The primary outcome measure was the SF-36 physical component summary (PCS). NSTI patients were compared according to intensive care unit (ICU) admission status. ICU survivors were matched on SAPS II with non-NSTI related septic shock survivors.

**Results:**

Forty-nine NSTI survivors were phone-interviewed and included in the study. Median PCS was decreased compared to the reference population [− 0.97 (− 2.27; − 0.08) SD]. Previous cardiac disease was the only variable associated with PCS alteration [multivariate regression coefficient: − 8.86 (− 17.64; − 0.07), *p*  =  0.048]. Of NSTI survivors, 15.2% had a HAD-D score  ≥  5 and 61.2% an IES-R score  ≥  33. ICU admission was not associated with lower PCS [35.21 (25.49–46.54) versus (vs) 41.82 (24.12–51.01), *p*  =  0.516], but with higher IES-R score [14 (7.5–34) vs 7 (3–18), *p*  =  0.035] and a higher proportion of HAD-D score  ≥  5 (28.6 vs 4.0%, *p*  =  0.036). Compared to non-NSTI septic shock-matched controls, NSTI patients had similar PCS [33.81 (24.58; − 44.39) vs 44.87 (26.71; − 56.01), *p * =  0.706] but higher HAD-D [3.5 (1–7) vs 3 (1.5–6), *p*  =  0.048] and IES-R scores [18 (8–35) vs 8 (3–19), *p*  =  0.049].

**Conclusions:**

Long-term QOL in NSTI survivors is severely impaired, similarly to that of non-NSTI septic shock patients for physical compartments, but with more frequent depressive and/or post-traumatic stress disorders. Only ICU admission and previous cardiac disease were predictive of QOL impairment.

**Supplementary Information:**

The online version contains supplementary material available at 10.1186/s13613-021-00891-9.

## Background

Necrotizing skin and soft-tissue infections (NSTIs) are a group of rare and severe infections associated with a high and stable mortality [1]. Intensive care unit (ICU) admission is frequently required. Urgent and aggressive surgical debridement of infected tissues is the mainstem of management [2–4], but is associated with severe sequelae among survivors, with 20% requiring amputations [5, 6].

Long-term quality-of-life (QOL) of NSTI survivors has been shown to be altered when compared to the global population [7–9], as has also been reported among ICU survivors overall [10–12]. Nevertheless, long-term QOL impairment of NSTI survivors is characterized by specific features such as chronic pain, sexual dysfunction in case of perineal involvement [13], or psychological distress due to changes in body appearance [8]. Comparing NSTI survivors to other ICU patients admitted for equally severe infections might thus allow for identifying specific needs during their follow-up. Moreover, admission characteristics, including the topographical characteristics of the infection, have not been associated with long-term QOL impairment [7, 13].

The aim of this study was to evaluate long-term quality of life in necrotizing soft-tissue infection survivors, compare it to an equally severe population of other infection survivors, and finally assess its association with admission characteristics.

## Methods

### Aim, patients and study design

The primary objective of the current study was to evaluate long-term QOL in NSTI survivors using validated psychological instruments [14–17]. Secondary objectives were to assess the impact of need for ICU admission and of other admission characteristics on QOL. In order to evaluate the severity of QOL impairment with a more comparable population than the global population, we conducted an exposed–unexposed cohort study matching NSTI patient to non-NSTI septic shock patients.

We conducted a prospective monocenter study including all consecutive adult patients who survived a hospital admission in our tertiary care center for NSTI between January 1st 2014 and September 29th 2017 [6]. NSTI survivors were categorized as “admitted to the ICU” in case of ICU admission for NSTI at any time during their hospital stay. Patients admitted to the ICU were subsequently matched 1:1 or 1:2 on SAPS II (±  5 points) to a cohort of non-NSTI septic shock survivors from a previous study assessing QOL [18].

Patients received information during their hospital stay that data abstracted from their medical charts could be used for research purposes. Data were anonymized and compiled according to the requirements of the Commission Nationale Informatique et Liberté (Registration Number 2003722) and the study was approved by the Comité de Protection des Personnes Ile-de-France V (Reference #16165). The study has been reported according to the STROBE guidelines regarding observational cohort studies.

### Phone interviews

Patients were contacted by an introductory letter explaining the nature of the study. Phone interviews were conducted from November 2017 to March 2018 by two dedicated nurses using an electronic standardized form.

### Data collection

The following data were collected: age, sex, main comorbidities, SAPS II, portal of entry of sepsis in patients with septic shock unrelated to NSTI, need for invasive mechanical ventilation, and hospital length of stay. To assess patient-reported outcomes, the following questionnaires were administered during phone interviews: (i) the validated French version of the Medical Outcome Study 36-Item Short-Form Health Survey (SF-36) questionnaire; (ii) the Hospital Anxiety and Depression (HAD) scale; (iii) the Impact of Event Scale-Revised (IES-R); (iv) the level of independence evaluated with the activities of daily living (ADL) and the instrumental activities of daily living (IADL) scales; (v) a self-assessed global quality of life evaluated with a quantitative score ranging from 0 (worst value) to 100 (best value); and (vi) a selection of relevant individual and contextual determinants of health-related QOL in this population.

Topographical characteristics of NSTIs were assessed by one investigator (TU) by retrospectively analyzing photographs of affected areas. These were available for 42 of the 49 patients, with post-operative photographs also available and used in priority for 30 patients. The following NSTI features were recovered: NSTI location (limb vs trunk and/or abdomino-perineal), articular skin involvement (yes or no) and circumferential skin necrosis/debridement (yes or no) for limb NSTIs. The percentage of body surface affected was also determined using the Wallace rule of nine adjusted by estimating the size of one palm to be 1% of total body surface [19].

### Outcome measures

The primary outcome measure was the Physical Component Summary (PCS) from the SF-36 questionnaire. The SF-36 is a validated, generic health-related quality of life questionnaire [20–22]. It comprises 36 items assessing eight health-related QOL dimensions, covering physical functioning, physical role, bodily pain, general health, vitality, social functioning, emotional role, and mental health. Physical and mental component summaries (PCS and MCS, respectively) constitute aggregates of the eight individual dimensions to provide summary scores from physically and mentally oriented subscales using weights updated for French population. The higher the PCS or MCS, the better the QOL.

### Statistical analysis

Quantitative data are presented as median [Interquartile range (IQR)] or mean  ±  standard deviations (SD), categorical data as number (percentages). Comparisons were made using the Student’s *t *test or the Mann–Whitney test for continuous variables, and the Chi-squared or Fisher exact tests for categorical variables, according to sample size.

QOL of NSTI survivors as a whole was first described using the crude values of QOL scores. SF-36 subdimensions were expressed as raw values (normalized on a 0–100 scale) and age-sex standardized values, using reference values of the French population [23], and expressed as SD (*z*-scores). SF-36 aggregate components (i.e*.*, PCS and MCS) were computed as recommended [24] and also expressed either on a normalized scale centered on 50 representing the population norm (i.e*.*, values  >  or  <  50 reflecting values higher or lower than age-sex standardized French values) or as age-sex standardized values (*z*-scores). The association of admission characteristics with PCS and MCS was assessed by linear regression. The final model was determined by entering all variables associated with the outcome at the *p*  <  0.10 level in univariate analysis. No backward elimination of the variables was performed. Results for continuous variables, assessed in univariate and multivariate analyses, were expressed per unit of the feature and regression coefficients [95% Confidence Interval (CI)] presented.

Exploratory analyses included comparisons between NSTI survivors admitted to the ICU and non-NSTI septic shock survivors matched on SAPS II, and comparisons between NSTI survivors who had or had not been admitted to the ICU. Analyses in the matched population were adjusted for age, sex and time elapsed between hospital discharge and phone interview, using mixed models.

Significance was defined as two-sided *p *value  <  0.05. Data were collected and entered into a Microsoft Excel 2010 (Microsoft Corporation, Redmond, WA) spreadsheet. All statistical analyses were performed using Stata v15.0 (StataCorp, College Station, TX, USA). Tables and figures were made using Stata v15.0, Microsoft Excel 2010 (Microsoft Corporation, Redmond, WA), and GraphPad Prism 5 (GraphPad Software Inc, La Jolla, Calif).

## Results

### Characteristics and long-term QOL of the whole study population

**Fig. 1:**
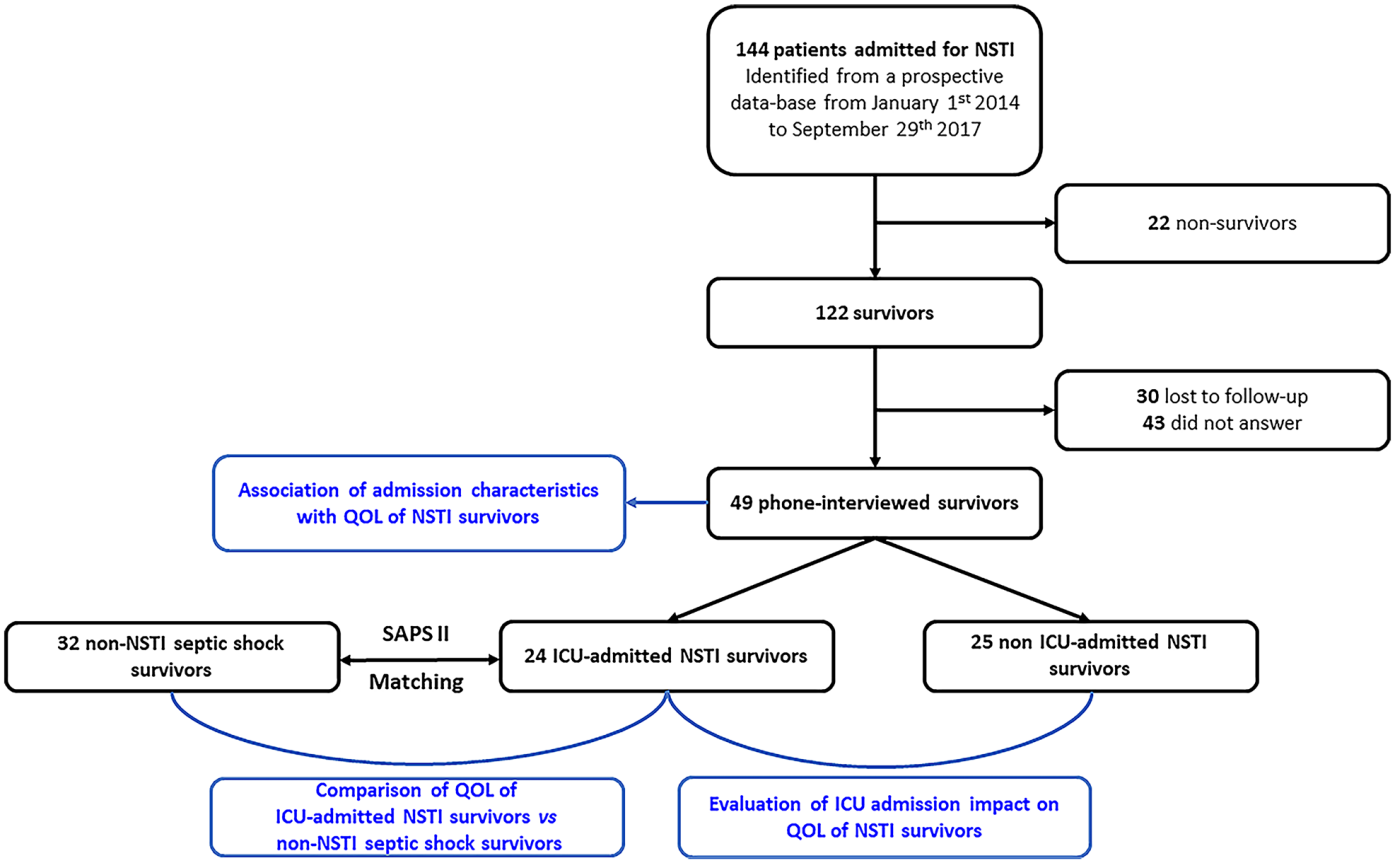
Study flowchart. Necrotizing soft tissue infection (NSTI) survivors (*n*  =  49) admitted (*n*  =  24) or not (*n*  =  25) to the intensive care unit (ICU) were included in the study. First, association of admission characteristics to the long-term health-related quality of life (QOL) of all NSTI survivors was assessed. Then, in order to explore the impact of both NSTI itself and of ICU admission on QOL, NSTI patients admitted to the ICU (*n*  =  24) were compared to: (1) non-ICU-admitted (*n*  =  25) NSTI patients; and (2) ICU-admitted non-NSTI septic shock patients (*n*  =  32) after SAPS II matching. The 24 NSTI patients admitted to the ICU were matched according to SAPS II on a 1:1 ratio for 10 patients and a 1:2 ratio for 11 patients, with 3 patients finding no match, to a total of 32 non-NSTI septic shock patients

From January 2014 to September 2017, 144 patients were admitted to our center for NSTI. Among these, 122 (84.7%) survived hospital discharge, of whom 30 were lost to follow-up, leaving 92 patients to be contacted. Of these, 43 did not answer the phone calls, leaving 49 patients who answered the QOL questionnaire after a median time period of 1.5 [1.1–2.4] years after hospital discharge, and were included in the study (Fig. 1).

Demographics, comorbidities, clinical features and QOL assessment of the study population are presented in Table 1. Patients were predominantly males with a median age of 60 [53–70] years, and the most frequent comorbidities were diabetes (36.7%), obesity (30.6%), cardiac disease (24.5%) and immunodeficiency (24.5%). Twenty-four patients (49.0%) were admitted to the ICU, of whom 11 required mechanical ventilation and 18 developed septic shock during the course of hospital stay. Regarding NSTI characteristics, limbs were the most commonly involved area (83.7%), while the trunk and/or the abdomino-perineal region were less frequently affected (18.4%). Median body surface affected was 4.0% [2.0–4.5] with 15.6% of circumferential lesions and 46.9% of articular involvement. Nine patients (18.4%) required limb amputations. QOL in NSTI survivors was severely impaired for physical components when compared to the global population, as assessed by the SF-36 with an age-sex standardized PCS aggregate score lower than that of the reference population [36.4 (24.4–48.6), *z*-score  =  − 0.97 (− 2.27; − 0.08) SD]. Regarding mental health, and although the MCS aggregate score of NSTI survivors was even slightly higher than that of the reference population [55.6 (48.8–62.9), *z*-score =   + 0.41 SD], 37.0% of patients had a HAD-D score  ≥  5 and 20.4% an IES-R score  ≥  33, pointing to a significant incidence of depressive and/or post-traumatic stress disorders in NSTI survivors.

**Table 1:** Demographics, comorbidities, clinical features and quality of life assessment of all necrotizing soft-tissue infection patients included

	Available data	All patients *n* = 49
Demographics		
Age, years, median [IQR]	49	60 [53–70]
Male gender		30 (61.2)
Comorbidities		
Diabetes mellitus	49	18 (36.7)
Immunodeficiency		12 (24.5)
Cancer		4 (8.2)
Corticosteroids		8 (16.3)
Obliterating arteritis of the lower limbs		6 (12.2)
Chronic kidney disease		3 (6.1)
Chronic obstructive pulmonary disease		6 (12.2)
Cardiac disease		12 (24.5)
Liver cirrhosis		1 (2.0)
Chronic alcohol consumption		5 (10.2)
Obesity		15 (30.6)
Clinical characteristics		
ICU admission	49	24 (49.0)
SAPS II^a^, median [IQR]	39	26 [18–37]
SOFA score^a^, median [IQR]	45	1 [0–7]
Mechanical ventilation	49	11 (22.5)
Shock^b^	47	18 (38.3)
Duration of hospital stay, days, median [IQR]	49	21 [12–32]
NSTI topography	49	
Limbs		41 (83.7)
Trunk and/or abdomino-perineal		9 (18.4)
Body surface affected, %; median [IQR]	42	4 [2–4.5]
Circumferential infection	45	7 (15.6)
Articular skin involvement	49	23 (46.9)
Number of surgical debridements, median [IQR]	49	1 [1, 2]
Quality of life assessment		
Time between discharge and interview, years; median [IQR]	49	1.5 [1.1–2.4]
Self-assessed global quality of life, median [IQR]	49	65 [50–80]
Functioning level/independence		
ADL, median [IQR]	48	6 [5.5–6]
IADL, median [IQR]	49	7 [5–8]
Mental health		
HAD-A, median [IQR]	49	5 [3–9]
HAD-A ≥ 8		14 (28.6)
HAD-D, median [IQR]	46	3 [1–6]
HAD-D ≥ 5		17 (37.0)
IES-R, median [IQR]	49	9 [5–22]
IES-R ≥ 33		10 (20.4)
General quality of life outcomes		
Current place of residence		
Sheltered housing	49	–
Care home		1 (2.0)
Private home without assistance		41 (83.7)
Private home with assistance		7 (14.3)
Sequelae related to NSTI		31 (63.3)
Amputations		9 (18.4)
Walking distance		
Not able to walk	49	2 (4.1)
< 50 m		5 (10.2)
50–200 m		6 (12.2)
200–500 m		4 (8.2)
> 500 m		32 (65.3)
Rehospitalizations	49	25 (51.0)
Current employment status		
Full-time employment/studies	49	10 (20.4)
Part-time employment/studies		2 (4.1)
Occasional employment		–
Unemployed		2 (4.1)
Retired		23 (46.9)
Long-term disability		12 (24.5)
Change in employment status since illness	49	18 (36.7)
Change in family status	49	6 (12.2)
In a relationship		1 (25.0)
Separated		3 (75.0)
Parenthood		1 (16.7)
Augmented alcohol consumption	49	1 (2.0)
Augmented tobacco consumption		1 (2.0)
Augmented drug consumption		0 (0.0)

Qualitative variables are shown as *n* (%)

*SAPS II *Simplified Acute Physiology Scale II; *SOFA* Sequential Organ Failure Assessment; *NSTI* necrotizing soft tissue infection; *ADL* Activities of Daily Living scale; *IADL* Instrumental Activities of Daily Living scale; *HAD-A* Hospital Anxiety and Depression scale-Anxiety; *HAD-D* Hospital Anxiety and Depression scale-Depression; *IES-R* Impact of Events Scale-Revised

^a^Within 24 h of hospital admission

^b^Defined as need for vasopressors during the course of intensive care unit (ICU) stay

### Association of admission characteristics with long-term QOL

An exploratory analysis found that after multivariate adjustment of admission characteristics, only previous cardiac disease remained associated with long-term physical QOL, as assessed by the PCS of the SF-36 [multivariate regression coefficient − 8.86 (− 17.64; − 0.07), *p*  =  0.048; Table 2]. Importantly, no topographical NSTI characteristic or other comorbidity was significantly associated with the PCS, although there was a tendency for circumferential infections [multivariate regression coefficient − 9.46 (− 19.66; 0.73), *p*  =  0.068], immunodeficiency [multivariate regression coefficient − 7.57 (− 16.53; 1.38), *p*  =  0.095] and obliterating arteritis [multivariate regression coefficient − 8.86 (− 21.65; 0.52), *p*  =  0.061] to be associated with lower PCS (i.e*.*, worst physical health). No admission characteristic was associated with long-term mental quality of life, as assessed by the MCS of the SF-36 (see Additional file 1: Table S1).

**Table 2:** Relationship between admission characteristics and quality of life as assessed by the PCS of the SF-36, for all necrotizing soft-tissue infection patients included

Admission characteristics	Available data	Univariate regression coefficient [95% CI]	Univariate *p *value	Multivariate regression coefficient [95% CI]	Multivariate *p *value
Age	48	− 0.36 [− 0.62; − 0.10]	**0.009**	− 0.21 [− 0.47; 0.06]	0.127
SAPS II	38	− 0.03 [− 0.31; 0.25]	0.823	–	–
SOFA score	44	− 0.04 [− 0.98; 0.90]	0.933	–	–
Sex	48				
Female (*n* = 19)		Ref.			
Male (*n * = 29)		2.17 [− 6.04; 10.39]	0.597	–	–
Diabetes mellitus	48				
No (*n* = 30)		Ref.			
Yes (*n* = 18)		− 3.05 [− 11.32; 5.22]	0.462	–	–
Immunodeficiency	48				
No (*n* = 36)		Ref.		Ref.	
Yes (*n* = 12)		− 7.71 [− 16.72; 1.31]	**0.092**	− 7.57 [− 16.53; 1.38]	0.095
Obliterating arteritis	48				
No (*n* = 42)		Ref.		Ref.	
Yes (*n* = 6)		− 10.37 [− 22.16; 1.41]	**0.083**	− 10.56 [− 21.65; 0.52]	0.061
Chronic kidney disease	48				
No (*n* = 45)		Ref.			
Yes (*n* = 3)		− 6.75 [− 23.28; 9.77]	0.415	–	–
Chronic alcohol consumption	48				
No (*n* = 43)		Ref.		–	–
Yes (*n* = 5)		− 4.50 [− 17.62; 8.62]	0.494	–	–
Obesity	48				
No (*n* = 33)		Ref.			
Yes (*n* = 15)		− 2.69 [− 11.34; 5.97]	0.535	–	–
Cardiac disease	48				
No (*n* = 37)		Ref.		Ref.	
Yes (*n* = 11)		− 9.83 [− 18.96; − 0.70]	**0.035**	− 8.86 [− 17.64; − 0.07]	**0.048**
COPD					
No (*n* = 42)		Ref.			
Yes (*n* = 6)		3.57 [− 8.56; 15.71]	0.556	–	–
ICU admission	48				
No (*n* = 25)		Ref.			
Yes (*n* = 23)		− 2.66 [− 10.68; 5.37]	0.508	–	–
Mechanical ventilation	48				
No (*n* = 38)		Ref.			
Yes (*n* = 10)		− 1.18 [− 11.09; 8.73]	0.812	–	–
Shock	46				
No (*n* = 29)		Ref.			
Yes (*n* = 17)		0.89 [− 7.06; 9.38]	0.833	–	–
Trunk and/or abdomino-perineal involvement	48				
No (*n* = 40)		Ref.			
Yes (*n* = 8)		− 2.15 [− 12.94; 8.68]	0.690	–	–
Circumferential infection	44				
No (*n* = 37)		Ref.		Ref.	
Yes (*n* = 7)		− 10.32 [− 21.72; 1.08]	**0.075**	− 9.46 [− 19.66; 0.73]	0.068
Articular skin involvement	48				
No (*n * = 25)		Ref.			
Yes (*n* = 23)		1.06 [− 7.00; 9.12]	0.792	–	–
Body surface affected	42	− 0.72 [− 2.34; 0.89]	0.372	–	–

Multivariate analysis for association with PCS included all variables with a *p *value  <  0.1 by univariate linear regression analysis. One patient who completed the questionnaire did not fully answer items needed for PCS calculation and only 48 patients were thus included

*COPD* chronic obstructive pulmonary disease; *SAPS II* Simplified Acute Physiology Scale II; *PCS* Physical Component Summary of the SF-36 questionnaire; *SF-36* 36-Item Short-Form Health Survey; *IQR* interquartile range; *CI* confidence interval; *SOFA* Sequential Organ Failure Assessment

^a^Within 24 h of hospital admission

^b^Defined as need for vasopressors during the course of intensive care unit (ICU) stay

Bolded *p* values are significant at the 0.05 level

### Comparison between NSTI patients admitted to the ICU and matched non-NSTI septic shock survivors

**Fig. 2:**
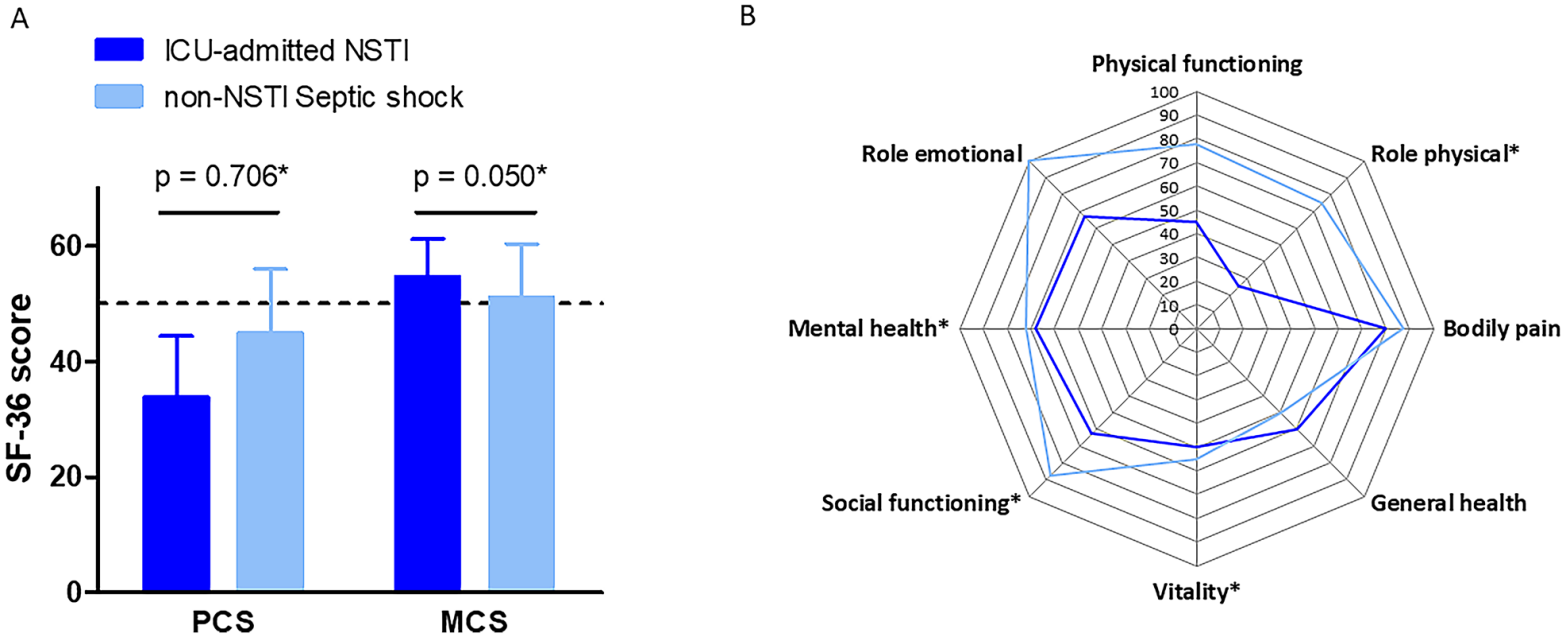
SF-36 questionnaire results in exposed (ICU-admitted NSTI survivors) and non-exposed (non-NSTI septic shock survivors) patients. **A** Comparison of the physical (PCS) and mental (PCS) component summaries of the SF-36 questionnaire between patients surviving necrotizing soft tissue infections (“ICU-admitted NSTI”, dark blue) and patients surviving a septic shock from other cause (“non-NSTI septic shock”, light blue). Results are centered on the value of 50 (dashed line; values  >  or  <  50 reflected values higher or lower than age-sex standardized French values). *p values come from a mixed logistic regression with adjustment for sex, SAPS II and the time between ICU discharge and phone interview, with pair as a random effect. **B** Comparison of each subdomain of the SF-36 questionnaire between patients surviving necrotizing soft tissue infections (“ICU-admitted NSTI”, dark blue) and patients surviving a septic shock from other cause (“non-NSTI septic shock”, light blue). The “physical functioning”, “role physical”, “bodily pain”, and “general health” subdimensions pertain to the PCS aggregate component, the “vitality”, “social functioning”, “mental health”, and “role emotional” subdimensions pertain to the MCS aggregate component. *Stands for statistical significance with *p* value  <  0.05 from comparing between groups the standard deviations of each score standardized for age and sex, by mixed logistic regression adjusted for sex, age and the time between ICU discharge and phone interview, with pair as a random effect. *NSTI* necrotizing soft-tissue infection; *MCS* mental component summary; *PCS* physical component summary; *SF-36* 36-Item Short-Form Health Survey

The 24 NSTI patients admitted to the ICU were matched according to SAPS II on a 1:1 ratio for 10 patients and a 1:2 ratio for 11 patients, to a total of 32 non-NSTI septic shock patients (see Additional file 2: Table S2). NSTI patients were not consistently in shock (76.2 vs 100%, *p*  =  0.007) but had longer hospital stays [29 (16–37) vs 7.5 (4–17.5) days, *p*  =  0.008] and shorter time elapsed between hospital discharge and phone interview than non-NSTI patients [1.1 (0.7–2.2) vs 4.5 (2.9–5.9) years, *p*  =  0.002]. Strikingly, NSTI patients had higher HAD-D scores [3.5 (1–7) vs 3 (1.5–6), *p*  =  0.048], and higher IES-R scores [18 (8–35) vs 8 (3–19), *p*  =  0.049] with a higher proportion of IES-R scores  ≥  335 (33.3 vs 6.3%, *p*  =  0.034). There were no differences regarding the self-assessed quality of life, as well as the ADL and IADL scores, the current place of residence, and the employment status or change in family status. There were no significant differences in the PCS [33.8 (24.6–44.4) vs 44.9 (26.7–56.0), *p*  =  0.706] and MCS [54.5 (44.5–61.2) vs 51.1 (44.7–60.4), *p*  =  0.050] aggregate scores between NSTI and non-NSTI ICU patients (Fig. 2A). Nevertheless, after standardization for age and sex, several subdimensions including role physical (*p*  =  0.016), vitality (*p*  =  0.033), social functioning (*p*  =  0.036), and mental health index (*p*  =  0.018) were significantly lower in NSTI patients when compared to their non-NSTI counterparts (Fig. 2B).

### Impact of ICU admission on quality of life

**Fig. 3:**
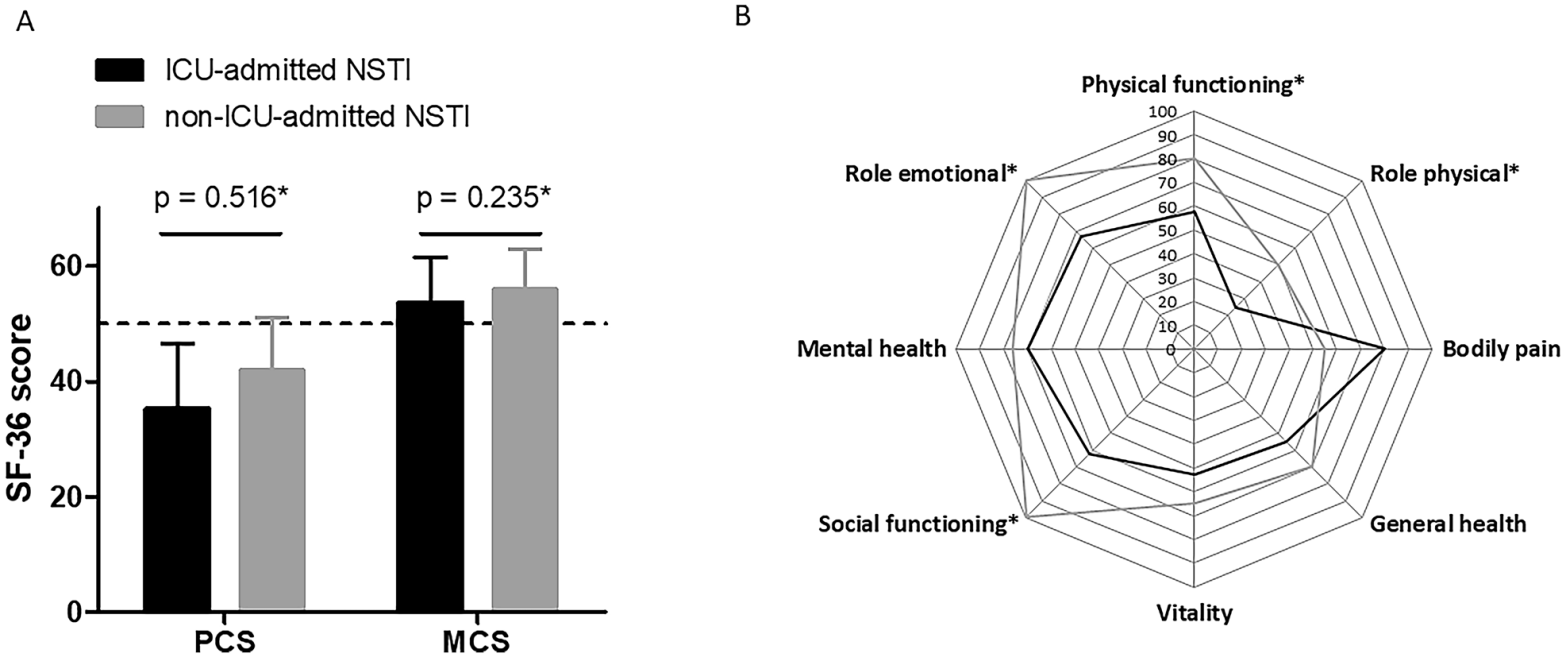
SF-36 questionnaire results in NSTI survivors admitted (black) or not admitted (gray) to the ICU. **A** Comparison of the physical (PCS) and mental (PCS) component summaries of the SF-36 questionnaire between patients surviving necrotizing soft tissue infections admitted (“ICU-admitted NSTI”, black) or non-admitted to the ICU (“non-ICU-admitted NSTI”, gray). Results are centered on the value of 50 (dashed line; values  >  or  <  50 reflected values higher or lower than age-sex standardized French values).*p values come from comparing between groups the standard deviations of each score standardized for age and sex. **B** Comparison of each subdomain of the SF-36 between patients surviving necrotizing soft tissue infections admitted (“ICU-admitted NSTI”, black) or non-admitted to the ICU (“non-ICU-admitted NSTI”, gray). The “Physical functioning”, “role physical”, “bodily pain”, and “general health” subdimensions pertain to the PCS aggregate component, the “vitality”, “social functioning”, “mental health”, and “role emotional” subdimensions pertain to the MCS aggregate component. *Stands for statistical significance with *p* value  <  0.05 from comparing between groups the standard deviations of each score standardized for age and sex. *MCS *mental component summary; *PCS* physical component summary; *SF-36* 36-Item Short-Form Health Survey; *ICU* intensive care unit

As expected, ICU patients showed significantly higher SAPS II scores than non-ICU patients [36 (27–52) vs 19 (16–42); *p*  <  0.01], but no significant differences regarding other admission characteristics. Patients who had been admitted to the ICU showed higher median IES-R score [14 (7.5–34) vs 7 (3–18), *p*  =  0.035], pointing to more frequent post-traumatic stress disorders (see Additional file 3: Table S3). Their employment status was also significantly different (*p*  =  0.023), with more frequent long-term disability (37.5 vs 12.0%) and less frequent full-time employment (8.3 vs 32.0%). Finally, they showed non-significant trends for lower self-assessed global QOL [50 (50–70) vs 70 (50–80), *p*  =  0.061], IADL [6 (4.5–8) vs 8 (6–8), *p*  =  0.072] and walking distances (*p*  =  0.059), together with higher HAD-A scores [6 (4.5–12) vs 4 (3–7), *p*  =  0.050]. There were no significant differences in the PCS [35.21 (25.49–46.54) vs 41.82 (24.12–51.01), *p * =  0.516] and MCS [53.62 (42.42–61.51) vs 55.85 (50.55–62.95), *p*  =  0.235] aggregate scores (Fig. 3A). Nevertheless, after standardization for age and sex, several subdimensions including physical functioning (*p*  =  0.014), role physical (*p * =  0.010), social functioning (*p*  =  0.005), and role emotional (*p*  =  0.031) were significantly lower in ICU than in non-ICU patients (Fig. 3B).

## Discussion

The main findings of this study are as follows: (1) long-term QOL in NSTI survivors was severely impaired when compared to that of the reference population, particularly for physical compartments; (2) no admission characteristic aside from previous cardiac disease, including topographical characteristics of the infection, were predictive of QOL impairment; (3) long-term QOL in NSTI survivors was relatively similar to that of non-NSTI septic shock survivors, expect for a trend towards more depressive and/or post-traumatic stress disorders; and (4) ICU admission was associated with a significant alteration in mental QOL in NSTI survivors.

Compared to the reference population, the QOL of NSTI survivors was severely impaired, particularly for physical compartments. Although a relatively preserved mental health was also noted in other works using the MCS of the SF-36 [25], we found, using a more extensive array of tools such as the HAD and IES scales, that mental health was more profoundly altered in NSTI survivors compared to non-NSTI septic shock-matched controls. Similarly, studies focusing on patient experience have revealed a severe psychosocial impact among NSTI survivors [26, 27].

We previously reported similar severe alteration of long-term QOL among both purpura fulminans and epidermal necrolysis survivors [18, 28]. The physical alteration among these patients was less severe than that of NSTI survivors when compared to the reference population (PCS SD, respectively, − 0.63 and − 0.44 vs − 1.59). There were no differences regarding mental health between purpura fulminans or epidermal necrolysis survivors and other septic shock patients, while for NSTI patients both HAD-D and IES-R scores were significantly higher, suggesting a more severe psychological distress in these patients. This could be due to the younger age of purpura fulminans and epidermal necrolysis patients [respectively, 43 (25–61) and 48 (35–59) years old in our previous works], as compared with NSTI patients [60 (53–70) years old for this cohort], who also have more comorbidities. This is in line with studies showing that older individuals, even among NSTI survivors, are more prone to alterations of their physical QOL, although they do not corroborate the finding that psychosocial adjustment could improve with age [29]. As for NSTI patients, ICU admission was associated with a more severely impaired QOL in epidermal necrolysis patients [28]. There was a trend for longer hospital stays in ICU-admitted compared to non-ICU admitted NSTI patients. Whether this longer, although non-significant, hospital stay or ICU admission in itself accounted for mental QOL impairment is unsettled [10]. Taken together, these results suggest that life-threatening conditions affecting the skin could be associated with a more severe long-term QOL impairment than other causes of septic shock, particularly in more frail and more severe patients.

We found no association between admission characteristics and QOL impairment aside from previous cardiac disease, including topographical characteristics of the infection. Although infections of the limbs have been associated with functional limitation [7], the latter has not been associated with impaired QOL, nor has articular involvement [30]. Moreover, specific fields of QOL could be affected for perineal infections such as sexual function [13]. Results regarding the association between the percentage of body surface area affected and QOL are conflicting [7, 8, 30]. Age, although only associated with PCS in univariate analysis in our study, likely because of its interdependence with the variable cardiac disease, has been shown to be the admission characteristic most consistently associated with long-term QOL in other studies [8, 9, 29–31].

The main limitations of the study are its monocentric and retrospective nature, with a relatively small sample size. Although the first may limit generalizability of our results, both admission characteristics and QOL measurement are in line with the literature [8, 9, 25, 30]. We cannot exclude that a lack of statistical power might have accounted for the absence of significant correlation between admission characteristics and long-term QOL, as well as of significant differences in QOL between NSTI and non-NSTI ICU survivors. Not all patients were admitted to the ICU, and few had perineal NSTI, which can be associated with specific alterations of QOL [13]. This warrants further larger scale multicenter studies. All ICU admitted NSTI patients were not in shock, but were matched on SAPS II to non-NSTI-related septic shock survivors to obtain more comparable populations in terms of severity, although residual confounders may remain, such as other unrecorded comorbidities. Responders and non-responders were comparable on sex (59 vs 51% male, *p*  =  0.440) and severity of illness as assessed by admission SAPS II [25 (18–36) vs 27 (20–34), *p*  =  0.441], but there was a non-significant trend for non-responders to be older [58 (52–69) vs 66 (53–78) years, *p*  =  0.074], and a selection bias is possible. Finally, we did not assess microbiological characteristics or therapeutic interventions and their association with QOL, although in a small-scale study *E. coli* infections and an important number of surgical excisions were predictors of poor outcome [30]. Similarly, marital status has been associated with QOL for NSTI survivors, an association we did not evaluate [8, 29].

Our results are supportive of a more intense long-term follow-up of NSTI patients including physiotherapy and psychological support to detect and manage both physical and mental alterations of QOL, particularly in patients whose condition was severe upon admission. These findings finally highlight the need for further research in the little explored field of NSTI, using patient-centered outcomes.

## Conclusion

Long-term QOL in NSTI survivors was severely impaired when compared to the reference population, particularly for physical compartments. It was relatively similar to that of non-NSTI septic shock patients, expect for a trend towards more frequent depressive and/or post-traumatic stress disorders. Apart from ICU admission and previous cardiac disease, no admission characteristic, including topographical characteristics of the infection, were predictive of QOL impairment.

## Supplementary Information

**Additional file 1: Table S1.** Relationship between admission characteristics and quality of life as assessed by the MCS of the SF-36, for all included NSTI survivors.

**Additional file 2: Table S2.** Demographics, comorbidities, clinical features and quality of life assessment of ICU admitted NSTI patients and matched non-NSTI septic shock ICU patients.

**Additional file 3: Table S3.** Demographics, comorbidities, clinical features and quality of life assessment of whole study population, NSTI patients admitted or not admitted to the ICU.

## Data Availability

The datasets used and/or analyzed during the current study are available from the corresponding author on reasonable request.

## Abbreviations

ADL: Activities of Daily Living
HAD: Hospital Anxiety and Depression Scale
HR: Hazard ratio
I-ADL: Instrumental Activities of Daily Living
ICU: Intensive care unit
IES-R: Impact of Events Scale-Revised
SAPS II: Simplified Acute Physiology Score II
SOFA: Sequential Organ Failure Assessment
MCS: Mental component summary
NSTI: Necrotizing soft tissue infection
PCS: Physical component summary
QOL: Quality of life
SD: Standard deviation
SF-36: 36-Item short-form health survey

## References

[1] Al-Qurayshi Z, Nichols RL, Killackey MT, Kandil E (2020). Mortality risk in necrotizing fasciitis: national prevalence, trend, and burden. Surg Infect.

[2] Stevens DL, Bisno AL, Chambers HF, Dellinger EP, Goldstein EJC, Gorbach SL (2014). Practice guidelines for the diagnosis and management of skin and soft tissue infections: 2014 update by the Infectious Diseases Society of America. Clin Infect Dis.

[3] Urbina T, Madsen MB, de Prost N (2020). Understanding necrotizing soft tissue infections in the intensive care unit. Intensiv Care Med.

[4] Peetermans M, de Prost N, Eckmann C, Norrby-Teglund A, Skrede S, De Waele JJ (2019). Necrotizing skin and soft-tissue infections in the intensive care unit. Clin Microbiol Infect.

[5] Madsen MB, Skrede S, Perner A, Arnell P, Nekludov M, INFECT Study Group (2019). Patient’s characteristics and outcomes in necrotising soft-tissue infections: results from a Scandinavian, multicentre, prospective cohort study. Intensiv Care Med.

[6] Urbina T, Hua C, Sbidian E, Bosc R, Tomberli F, Lepeule R (2019). Impact of a multidisciplinary care bundle for necrotizing skin and soft tissue infections: a retrospective cohort study. Ann Intensiv Care.

[7] Pham TN, Moore ML, Costa BA, Cuschieri J, Klein MB (2009). Assessment of functional limitation after necrotizing soft tissue infection. J Burn Care Res.

[8] Gawaziuk JP, Strazar R, Cristall N, Logsetty S (2018). Factors predicting health-related quality of life following necrotizing fasciitis. J Plast Reconstr Aesthet Surg.

[9] Kruppa C, Hutter DJ, Königshausen M, Gessmann J, Schildhauer TA, Coulibaly MO (2019). Necrotizing fasciitis and the midterm outcomes after survival. SAGE Open Med.

[10] Ehooman F, Biard L, Lemiale V, Contou D, de Prost N, Mokart D (2019). Long-term health-related quality of life of critically ill patients with haematological malignancies: a prospective observational multicenter study. Ann Intensive Care.

[11] Nannan Panday RS, Minderhoud TC, Chantalou DS, Alam N, Nanayakkara PWB (2019). Health related quality of life in sepsis survivors from the prehospital antibiotics against sepsis (PHANTASi) trial. PLOS ONE.

[12] Apfelbacher C, Brandstetter S, Blecha S, Dodoo-Schittko F, Brandl M, The DACAPO Study Group (2020). Influence of quality of intensive care on quality of life/return to work in survivors of the acute respiratory distress syndrome: prospective observational patient cohort study (DACAPO). BMC Public Health.

[13] Czymek R, Kujath P, Bruch H-P, Pfeiffer D, Nebrig M, Seehofer D (2013). Treatment, outcome and quality of life after Fournier’s gangrene: a multicentre study. Colorectal Dis.

[14] Katz S (1963). Studies of illness in the aged: the index of ADL: a standardized measure of biological and psychosocial function. JAMA.

[15] Lawton MP, Brody EM (1970). Assessment of older people: self-maintaining and instrumental activities of daily living. Nurs Res.

[16] Zigmond AS, Snaith RP (1983). The Hospital Anxiety and Depression Scale. Acta Psychiatr Scand.

[17] Weiss DS, Marmar CR, Wilson JP, Keane TM (1997). The impact of event scale—revised. Assessing psychological trauma and PTSD.

[18] Contou D, Canoui-Poitrine F, Coudroy R, Préau S, Cour M, Barbier F (2019). Long-term quality of life in adult patients surviving Purpura fulminans: an exposed-unexposed multicenter cohort study. Clin Infect Dis.

[19] American College of Surgeons, Committee on Trauma (2018). Advanced trauma life support: student course manual.

[20] McHorney CA, Ware JE, Raczek AE (1993). The MOS 36-Item Short-Form Health Survey (SF-36): II. Psychometric and clinical tests of validity in measuring physical and mental health constructs. Med Care.

[21] Contopoulos-Ioannidis DG, Karvouni A, Kouri I, Ioannidis JPA (2009). Reporting and interpretation of SF-36 outcomes in randomised trials: systematic review. BMJ.

[22] Torrance N, Smith BH, Lee AJ, Aucott L, Cardy A, Bennett MI (2009). Analysing the SF-36 in population-based research. A comparison of methods of statistical approaches using chronic pain as an example. J Eval Clin Pract.

[23] Leplège A, Ecosse E, Verdier A, Perneger TV (1998). The French SF-36 Health Survey: translation, cultural adaptation and preliminary psychometric evaluation. J Clin Epidemiol.

[24] Ware JE, Sherbourne CD (1992). The MOS 36-item short-form health survey (SF-36). I. Conceptual framework and item selection. Med Care.

[25] Suijker J, de Vries A, de Jong VM, Schepers T, Ponsen KJ, Halm JA (2020). Health-related quality of life is decreased after necrotizing soft-tissue infections. J Surg Res.

[26] Hakkarainen TW, Burkette Ikebata N, Bulger E, Evans HL (2014). Moving beyond survival as a measure of success: understanding the patient experience of necrotizing soft-tissue infections. J Surg Res.

[27] Fagerdahl A-M, Knudsen VE, Egerod I, Andersson AE (2020). Patient experience of necrotising soft-tissue infection from diagnosis to six months after intensive care unit stay: a qualitative content analysis. Aust Crit Care.

[28] Ingen-Housz-Oro S, Alves A, Colin A, Ouedraogo R, Layese R, Canoui-Poitrine F (2020). Health-related quality of life and long-term sequelae in survivors of epidermal necrolysis: an observational study of 57 patients. Br J Dermatol.

[29] Pikturnaite J, Soldin M (2014). Impact of necrotising fasciitis on quality of life: a qualitative analysis. Burns.

[30] Chevet-Noël A, Andreoletti JB, Kheloufi M, Pluvy I (2020). Atteinte des membres dans les DHBN-FN : étude bicentrique entre 2000 et 2017 sur la qualité de vie et impact fonctionnel. Ann Chir Plast Esthét.

[31] Brengard-Bresler T, De Runz A, Bourhis F, Mezzine H, Khairallah G, Younes M (2017). Postoperative quality of life of patients with a bacterial necrotizing dermis-hypodermitis or necrotizing fasciitis, a ten-year study. Ann Chir Plast Esthet.

