# Supplementary material for: Long-term quality of life in necrotizing soft-tissue infection survivors: a monocentric prospective cohort study
Source: Ann Intensive Care. 2021 Jul 2;11:102. doi: 10.1186/s13613-021-00891-9 (PMC8253876; doi:10.1186/s13613-021-00891-9)
Supplement: Supplementary file 1 — Additional file 1: Table S1. Relationship between admission characteristics and quality of life as assessed by the MCS of the SF-36, for all included NSTI survivors. [file 13613_2021_891_MOESM1_ESM.docx]

**Table S1**. **Relationship between admission characteristics and quality of life as assessed by the MCS of the SF-36, for all included NSTI survivors.** As no variable reached a significant univariate association with MCS, no multivariable analysis was performed. One patient who completed the questionnaire did not fully answer items needed for MCS calculation and only 48 patients were thus included.

| **Admission characteristics** | **Available  data** | **MCS (median [IQR])** | **Univariate regression coefficient [95% CI]** | **Univariate P-value** |
| --- | --- | --- | --- | --- |
| **Age** | 48 | - | 0.10 [-0.18 ; 0.38] | 0.464 |
| **SAPS II** | 38 | - | -0.07 [-0.34 ; 0.19] | 0.578 |
| **SOFA score** | 44 | - | -0.47 [-1.43 ; 0.49] | 0.329 |
| **Sex** | 48 |  |  |  |
| Female (n=19) |  | 55.7 [50.6 – 62.7] | 5.12 [-2.80 ; 13.04] | 0.200 |
| Male (n=29) |  | 53.9 [36.0 – 62.9] | Ref |  |
| **Diabetes mellitus** | 48 |  |  |  |
| No (n=30) |  | 54.7 [42.4 – 62.7] | Ref |  |
| Yes (n=18) |  | 56.4 [50.6 – 65.3] | 4.20 [-3.85 ; 12.25] | 0.299 |
| **Immune deficiency** | 48 |  |  |  |
| No (n=36) |  | 55.2 [47.0 – 62.8] | Ref |  |
| Yes (n=12) |  | 55.6 [51.2 – 64.1] | 1.97 [-7.12 ; 11.06] | 0.665 |
| **Obliterating arteritis** | 48 |  |  |  |
| No (n=42) |  | 55.1 [47.4 – 62.9] | Ref |  |
| Yes (n=6) |  | 56.4 [52.3 – 60.8] | 1.79 [-10.12 ; 13.70] | 0.764 |
| **Chronic Kidney disease** | 48 |  |  |  |
| No (n=45) |  | 54.7 [47.4 – 62.9] | Ref |  |
| Yes (n=3) |  | 60.8 [55.7 – 66.3] | 8.16 [-7.95 ; 24.27] | 0.313 |
| **Chronic alcohol consumption** | 48 |  |  |  |
| No (n=43) |  | 55.7 [47.4 – 62.9] | Ref |  |
| Yes (n=5) |  | 53.5 [53.5 – 57.1] | 0.68 [-12.23 ; 13.59] | 0.916 |
| **Obesity** | 48 |  |  |  |
| No (n=33) |  | 56.0 [50.5 – 62.9] | Ref |  |
| Yes (n=15) |  | 53.5 [46.5 – 55.8] | -3.44 [-11.88 ; 5.01] | 0.417 |
| **Cardiac disease** | 48 |  |  |  |
| No (n=37) |  | 38.5 [27.5 – 50.1] | Ref |  |
| Yes (n=11) |  | 24.1 [19.6 – 44.6] | 1.12 [-8.26 ; 10.50] | 0.811 |
| **Chronic obstructive pulmonary disease** | 48 |  |  |  |
| No (n=42) |  | 35.6 [24.1 – 47.4] | Ref |  |
| Yes (n=6) |  | 47.6 [25.5 – 50.1] | -4.64 [-16.48 ; 7.20] | 0.434 |
| **ICU admission** | 48 |  |  |  |
| No (n=25) |  | 55.9 [50.5 – 62.9] | Ref |  |
| Yes (n=23) |  | 53.6 [42.4 – 61.5] | -6.05 [-13.74 ; 1.63] | 0.120 |
| **Mechanical ventilation** | 48 |  |  |  |
| No (n=38) |  | 55.6 [46.5 – 62.9] | Ref |  |
| Yes (n=10) |  | 54.8 [51.8 – 60.9] | 2.50 [-7.18 ; 12.18] | 0.606 |
| **Shock** | 46 |  |  |  |
| No (n=29) |  | 55.8 [47.4 – 62.9] | Ref |  |
| Yes (n=17) |  | 53.6 [50.6 – 60.9] | -3.81 [-12.18 ; 4.56] | 0.365 |
| **Trunk and/or abdomino-perineal involvement** | 48 |  |  |  |
| No (n=40) |  | 55.8 [48.8 – 62.9] | Ref |  |
| Yes (n=8) |  | 51.3 [41.9 – 59.3] | -4.98 [-15.46 ; 5.50] | 0.344 |
| **Circumferential infection** | 44 |  |  |  |
| No (n=37) |  | 55.4 [50.1 – 62.7] | Ref |  |
| Yes (n=7) |  | 57.1 [53.6 – 66.8] | 5.95 [-4.85 ; 16.74] | 0.273 |
| **Articular skin involvement** | 48 |  |  |  |
| No (n=25) |  | 55.4 [50.7 – 62.9] | Ref |  |
| Yes (n=23) |  | 55.9 [42.4 – 62.7] | -1.41 [-9.30 ; 6.47] | 0.720 |
| **Body surface affected** | 42 | - | 0.50 [-1.00 ; 2.00] | 0.505 |

SAPS II, Simplified Acute Physiology Scale II; SOFA, Sequential Organ Failure Assessment ; ICU, Intensive Care Unit
