# Supplementary material for: Long-term quality of life in necrotizing soft-tissue infection survivors: a monocentric prospective cohort study
Source: Ann Intensive Care. 2021 Jul 2;11:102. doi: 10.1186/s13613-021-00891-9 (PMC8253876; doi:10.1186/s13613-021-00891-9)
Supplement: Supplementary file 2 — Additional file 2: Table S2. Demographics, comorbidities, clinical features and quality of life assessment of ICU admitted NSTI patients and matched non-NSTI septic shock ICU patients. [file 13613_2021_891_MOESM2_ESM.docx]

**Table S2**. **Demographics, comorbidities, clinical features and quality of life assessment of intensive care unit (ICU)-admitted necrotizing soft tissue infection (NSTI) patients and matched non-NSTI septic shock ICU patients.** Matching on SAPS II was performed with a 1:1 or 1:2 ratio.

|  | **Available data** | **ICU NSTI patients**  **n=21** | **Septic shock^a^ patients**  **n=32** | **p-value^b^** |  |
| --- | --- | --- | --- | --- | --- |
| **Demographics** | | | | | |
| Age, years, median [IQR] | | 53 | 58 [53 – 71] | 50.5 [37 – 64.5] | 0.234 |
| Male gender, n (%) | |  | 15 (71.4) | 9 (28.1) | 0.084 |
| **Severity** | | | | | |
| SAPS II, median [IQR] | | 53 | 33 [26 – 52] | 31 [26.5 – 47] | 0.490 |
| Mechanical ventilation, n (%) | | 53 | 10 (47.6) | 22 (68.8) | 0.362 |
| Shock, n (%) | | 53 | 16 (76.2) | 32 (100%) | **0.007** |
| Duration of hospital stay, days [IQR] | | 53 | 29 [16 – 37] | 7.5 [4 – 17.5] | **0.008** |
| **Quality of life assessment** | | | | | |
| Time between discharge and interview, years, median [IQR] | | 53 | 1.1 [0.7 – 2.2] | 4.5 [2.9 – 5.9] | **0.002** |
| Self-assessed global quality of life, median [IQR] | | 53 | 50 [50 – 70] | 70 [50 – 80] | 0.065 |
| Functioning level/independence | | | | | |
| ADL, median [IQR] | | 52 | 5.5 [5 – 6] | 6 [5.5 – 6] | 0.230 |
| IADL, median [IQR] | | 50 | 6 [4 – 7] | 7 [7 – 8] | 0.061 |
| Mental Health | | | | | |
| HAD-A, median [IQR] | | 53 | 6 [5 – 12] | 7 [4.5 – 9.5] | 0.098 |
| HAD-A ≥ 8 | |  | 8 (38.1) | 13 (40.6) | 0.447 |
| HAD-D, median [IQR] | | 50 | 3.5 [1 – 7] | 3 [1.5 – 6] | **0.048** |
| HAD-D ≥ 5 | |  | 8 (44.4) | 13 (40.6) | 0.221 |
| IES-R, median [IQR] | | 53 | 18 [8 – 35] | 8 [3 – 19] | **0.049** |
| IES-R ≥ 33 | |  | 7 (33.3) | 2 (6.3) | **0.034** |
| General quality of life outcomes | | | | | |
| Current place of residence | |  |  |  | >0.999 |
| Sheltered housing | |  | - | - |  |
| Care home | |  | 1 (4.8) | - |  |
| Private home without assistance | |  | 15 (71.4) | 27 (84.4) |  |
| Private home with assistance | |  | 5 (23.8) | 5 (15.6) |  |
| Current employment status | | | | | |
| Full-time employment/studies | | 53 | 1 (4.7) | 5 (15.6) | - |
| Part-time employment/studies | |  | - | 4 (12.5) | - |
| Occasional employment | |  | - | 1 (3.1) | - |
| Unemployed | |  | 2 (9.5) | 4 (12.5) | - |
| Retired | |  | 9 (42.9) | 10 (31.3) | - |
| Long-term disability | |  | 9 (42.9) | 8 (25.0) | - |
| Change in family status | | 53 | 4 (19.1) | 3 (9.4) | - |
| In a relationship | |  | 1 (33.3) | 1 (33.3) | - |
| Separated | |  | 2 (66.7) | 2 (66.7) | - |
| Parenthood | |  | 1 (25.0) | 1 (33.3) | - |

^a^ Patients with septic shock had the following portal of entry: respiratory tract (n=19), urinary tract (n=5), non-necrotizing skin and soft tissue infections/indwelling catheters infections (n=6), others (n=2); ^b^ P-values were calculated after adjustment for for age,sex and time elapsed between hospital discharge and phone interview; **bolded** p-values are significant at the 0.05 level ; SAPS II, Simplified Acute Physiology Scale II; ADL, Activities of Daily Living scale ; IADL, Instrumental Activities of Daily Living scale ; HAD-A, Hospital Anxiety and Depression scale - Anxiety ; HAD-D, Hospital Anxiety and Depression scale - Depression ;IES-R, Impact of Events Scale -Revised
