# Supplementary material for: Long-term quality of life in necrotizing soft-tissue infection survivors: a monocentric prospective cohort study
Source: Ann Intensive Care. 2021 Jul 2;11:102. doi: 10.1186/s13613-021-00891-9 (PMC8253876; doi:10.1186/s13613-021-00891-9)
Supplement: Supplementary file 3 — Additional file 3: Table S3. Demographics, comorbidities, clinical features and quality of life assessment of whole study population, NSTI patients admitted or not admitted to the ICU. [file 13613_2021_891_MOESM3_ESM.docx]

**Table S3**. **Demographics, comorbidities, clinical features and quality of life assessment of whole study population, necrotizing soft tissue infection (NSTI) patients admitted or not admitted to the intensive care unit (ICU).** P-values for univariate analysis between Non-ICU and ICU patients.

|  | **Available data** | **All patients**  **n= 49** | **Non-ICU patients n=25** | **ICU patients n=24** | **p-value** |  |
| --- | --- | --- | --- | --- | --- | --- |
| **Demographics** | | | | | | |
| Age, years, median [IQR] | | 49 | 60 [53 – 70] | 63 [55 – 70] | 59 [53 – 70.5] | 0.674 |
| Male gender, n (%) | |  | 30 (61.2) | 13 (52.0) | 17 (70.8) | 0.176 |
| **Comorbidities**, n (%) | | | | | | |
| Diabetes mellitus | | 49 | 18 (36.7) | 6 (24.0) | 12 (50.0) | 0.059 |
| Immunodeficiency | |  | 12 (24.5) | 6 (24.0) | 6 (25.0) | 0.935 |
| Cancer | |  | 4 (8.2) | 1 (4.0) | 3 (12.5) | 0.349 |
| Corticosteroids | |  | 8 (16.3) | 4 (16.0) | 4 (16.7) | 0.99 |
| Obliterating arteritis of the lower limbs | |  | 6 (12.2) | 2 (8.0) | 4 (16.7) | 0.417 |
| Chronic kidney disease | |  | 3 (6.1) | 1 (4.0) | 2 (8.3) | 0.609 |
| Chronic obstructive pulmonary disease | |  | 6 (12.2) | 4 (16.0) | 2 (8.3) | 0.667 |
| Cardiac disease | |  | 12 (24.5) | 8 (32.0) | 4 (16.7) | 0.321 |
| Liver cirrhosis | |  | 1 (2.0) | 1 (4.0) | 0 (0.0) | >0.99 |
| Chronic alcohol consumption | |  | 5 (10.2) | 2 (8.0) | 3 (12.5) | 0.667 |
| Obesity | |  | 15 (30.6) | 5 (20.0) | 10 (41.7) | 0.100 |
| **Severity** | | | | | | |
| SAPS II, median [IQR] | | 39 | 26 [18 – 37] | 19 [16 – 24] | 36 [27 – 52] | **<0.001** |
| SOFA, median [IQR] | | 45 | 1 [0 – 7] | 0 [0 – 3] | 6 [2 – 9] | **<0.001** |
| Mechanical ventilation, n (%) | | 49 | 11 (22.5) | 0 | 11 (45.8) | - |
| Shock, n (%) | | 47 | 18 (38.3) | 0 | 18 (75.0) | - |
| Duration of hospital stay, days [IQR] | | 49 | 21 [12 – 32] | 18 [13 – 25] | 27 [11.5 – 37] | 0.234 |
| Number of surgical debridements, n (%) | | 49 | 1 [1 - 2] | 1 [1 – 2] | 1 [1 – 2] | 0.255 |
| **NSTI topography** | | | | | | |
| Limbs, n (%) | | 49 | 41 (83.7) | 23 (92.0) | 18 (75.0) | 0.138 |
| Trunk and/or abdomino-perineal, n (%) | |  | 9 (18.4) | 2 (8.0) | 7 (29.2) | 0.074 |
| Body surface affected, %, median [IQR] | | 42 | 4 [2 – 4.5] | 3.5 [2 – 4] | 4.5 [3 – 8] | 0.183 |
| Circumferential infection, n (%) | | 45 | 7 (15.6) | 4 (16.0) | 3 (15.0) | >0.99 |
| Articular skin involvement, n (%) | | 49 | 23 (46.9) | 13 (52.0) | 10 (41.7) | 0.469 |
| **Quality of life assessment** | | | | | | |
| Time between discharge and interview, years, median [IQR] | | 49 | 1.5 [1.1 – 2.4] | 1.5 [1.3 – 2.6] | 1.3 [0.7 – 2.1] | 0.379 |
| Self-assessed global quality of life, median [IQR] | | 49 | 65 [50 – 80] | 70 [50 – 80] | 50 [50 – 70] | 0.061 |
| Functioning level/independence | | | |  |  |  |
| ADL, median [IQR] | | 48 | 6 [5.5 – 6] | 6 [5.5 – 6] | 6 [5 – 6] | 0.339 |
| IADL, median [IQR] | | 49 | 7 [5 – 8] | 8 [6 – 8] | 6 [4.5 – 8] | 0.072 |
| Mental Health | | | |  |  |  |
| HAD-A, median [IQR] | | 49 | 5 [3 – 9] | 4 [3 – 7] | 6 [4.5 – 12] | 0.050 |
| HAD-A ≥ 8 | |  | 14 (28.6) | 5 (20.0) | 9 (37.5) | 0.175 |
| HAD-D, median [IQR] | | 46 | 3 [1 – 6] | 3 [1 – 5] | 4 [2 – 8] | 0.202 |
| HAD-D ≥ 5 | |  | 17 (37.0) | 7 (28.0) | 10 (47.6) | 0.170 |
| IES-R, median [IQR] | | 49 | 9 [5 – 22] | 7 [3 – 18] | 14 [7.5 – 34] | **0.035** |
| IES-R ≥ 33 | |  | 10 (20.4) | 3 (12.0) | 7 (29.2) | 0.136 |
| General quality of life outcomes | | | |  |  |  |
| Current place of residence | | | |  |  | 0.165 |
| Sheltered housing | | 49 | - | - | - |  |
| Care home | |  | 1 (2.0) | 0 | 1 (4.2) |  |
| Private home without assistance | |  | 41 (83.7) | 23 (92.0) | 18 (75.0) |  |
| Private home with assistance | |  | 7 (14.3) | 2 (8.0) | 5 (20.8) |  |
| Sequelae related to NSTI | |  | 31 (63.3) | 17 (68.0) | 14 (58.3) | 0.483 |
| Amputations | |  | 9 (18.4) | 3 (12.0) | 6 (25.0) | 0.289 |
| Walking distance | | | |  |  | 0.059 |
| Not able to walk | | 49 | 2 (4.1) | 0 | 2 (8.3) |  |
| <50 m | |  | 5 (10.2) | 3 (12.0) | 2 (8.3) |  |
| 50–200 m | |  | 6 (12.2) | 1 (4.0) | 5 (20.8) |  |
| 200–500 m | |  | 4 (8.2) | 4 (16.0) | 0 |  |
| >500 m | |  | 32 (65.3) | 17 (68.0) | 15 (62.5) |  |
| Rehospitalizations | | 49 | 25 (51.0) | 13 (52.0) | 12 (50.0) | 0.889 |
| Current employment status | | | |  |  | **0.023** |
| Full-time employment/studies | | 49 | 10 (20.4) | 8 (32.0) | 2 (8.3) |  |
| Part-time employment/studies | |  | 2 (4.1) | 2 (8.0) | 0 |  |
| Occasional employment | |  | - | - | - |  |
| Unemployed | |  | 2 (4.1) | 0 | 2 (8.3) |  |
| Retired | |  | 23 (46.9) | 12 (48.0) | 11 (45.8) |  |
| Long-term disability | |  | 12 (24.5) | 3 (12.0) | 9 (37.5) |  |
| Change in employment status since illness | | 49 | 18 (36.7) | 7 (28.0) | 11 (45.8) | 0.196 |
| Change in family status | | 49 | 6 (12.2) | 1 (4.0) | 5 (20.8) | 0.098 |
| In a relationship | |  | 1 (25.0) | - | 1 (25.0) |  |
| Separated | |  | 3 (75.0) | - | 3 (75.0) |  |
| Parenthood | |  | 1 (16.7) | 0 | 1 (20.0) |  |
| Augmented alcohol consumption | | 49 | 1 (2.0) | 1 (2.0) | 0 | 1 (4.2) |
| Augmented tobacco consumption | |  | 1 (2.0) | 1 (2.0) | 0 | 1 (4.2) |
| Augmented drug consumption | |  | - | - | - | - |

SAPS II, Simplified Acute Physiology Scale II; SOFA, sequential organ failure assessment ; NSTI, Necrotizing Soft Tissue Infection; ADL, Activities of Daily Living scale ; IADL, Instrumental Activities of Daily Living scale ; HAD-A, Hospital Anxiety and Depression scale - Anxiety ; HAD-D, Hospital Anxiety and Depression scale - Depression ;IES-R, Impact of Events Scale –Revised; **bolded** p-values are significant at the 0.05 level.
